# Living with outpatient management as spouse to intensively treated acute leukemia patients

**DOI:** 10.1371/journal.pone.0216821

**Published:** 2019-05-15

**Authors:** Lene Østergaard Jepsen, Lone Smidstrup Friis, Dorte Gilså Hansen, Claus Werenberg Marcher, Mette Terp Høybye

**Affiliations:** 1 Department of Hematology, Odense University Hospital, Odense, Denmark; 2 Department of Hematology, National University Hospital, Copenhagen, Denmark; 3 Research Unit of General Practice, University of Southern Denmark, Odense, Denmark; 4 Interdisciplinary Research Unit, Elective Surgery Center, Silkeborg Regional Hospital, Silkeborg, Denmark; University of Auckland, NEW ZEALAND

## Abstract

**Background:**

Spouses have a key position in the treatment of patients with acute leukemia (AL) who are increasingly managed in an outpatient setting. Patients live at home but appear at the hospital every second day for follow-up visits. Patients must adhere to specific precautions due to an impaired immune system, which challenges and influence the life of the whole family. This qualitative study, based on individual and group interviews with spouses to AL patients in curative intended treatment, elucidates how the intense and substantial caregiver role affects the everyday lives of spouses to AL patients in curative intended treatment.

**Methods:**

Qualitative semi-structured group interviews (n = 6) and individual interviews (n = 5) with spouses to AL patients were conducted at different time points during the whole course of treatment. Theories of everyday life served as the theoretical framework.

**Results:**

The spouses described their life as a constant state of vigilance and attention as a consequence of the responsibility they felt arising from the treatment in the outpatient setting. These made them experience their role as a burden. The social life of the spouses and the families suffered substantially due to the precautions that were instated in the home. However, many experienced that relations in the family were developed positively.

**Conclusions:**

Close relatives experience additional psychosocial burdens instigated by the outpatient management regimens. This is important knowledge for the health care system to include in future development of AL outpatient settings, to prioritize and support offers to the relatives that recognize their sense of burden. This could apply not only to relatives of AL patients but to the relatives of other severely ill patients as well.

## Introduction

Close relatives have a key position in the treatment of patients with acute leukemia (AL) who are increasingly managed in an outpatient setting (OPS) as alternative to a long-lasting inpatient treatment. How this influences the everyday life and psychosocial well-being of the closest relatives is important to future treatment guidelines but is very sparsely described. For months AL patients live at home but appear at the hospital every second day for follow-up visits [1]. AL patients differ from most other cancer patients by being highly vulnerable to infections and severe bleedings for three to five weeks after each cycle of chemotherapy due to a very deep immunodeficiency [2]. All family members therefore have to live in accordance with a range of safety precautions. Adaption to the often rapidly changing health status of the patients is furthermore a challenge for the close relatives of cancer patients in general and comes into conflict with planning and maintaining everyday life [3, 4].

Empowerment within the caregiver role is dependent on the ability to assume responsibility and on being treated respectfully by professionals [5]. A challenging balance for close relatives between managing their own needs and meeting the needs for support and care of the patient, has been pointed out [6]. Other studies with close relatives to cancer patients reported a higher average number of concerns than patients themselves; concerns which focused on family issues and unmet needs regarding information on medical treatment and supportive care for the patient [7–9].

Since these substantial organizational changes of treatment settings were implemented only few years ago, the research focus has been on morbidity and safety. Very little evidence exists regarding how the everyday life of the closest relatives is affected. To address such knowledge gap this study aims to elucidate how the intense and substantial caregiver role affects the everyday lives of spouses to AL patients in curative intended treatment in an OPS.

## Materials and methods

### Design

This article is based on data from semi-structured individual and group interviews with relatives of AL patients in curative treatment conducted as a part of a larger qualitative study. The larger study combined participant observation in the OPS of the department, individual patient interviews at three different times, and group or individual interviews with their relatives [10]. Interviews were used to obtain insight into people’s life situation and experiences [11]. The inductive analysis presented here focused on exploring everyday life during AL outpatient management (OPM) in the perspective of the relatives based on the interviews with relatives.

### Participants

Invited participants were Danish speaking relatives to intensively curative intended treated AL patients managed in the OPS at the Department of Hematology, Odense University Hospital. No exclusion criteria were applied. A relative was defined as the person living with the patient during OPM, and who in collaboration with the patient, was responsible for meeting the obligations of the OPS. Relatives were invited to participate in the study after agreement with the patient. Eleven relatives, who all turned out to be spouses, were included consecutively from March to November 2014 (S1 Fig). Demographic characteristics are shown in Table 1.

**Table 1:** Demographic characteristics of relatives (n = 11).

Sample characteristics	Description
*Gender*	
Male	3 (27%)
Female	8 (73%)
*Age (years)*	
< 60	7 (64%)
≥ 60	4 (36%)
*Relationship to the patient*	
Husband	3 (27%)
Wife	8 (73%)
*Families with children (years)*	
< 18	2 (18%)
≥ 18	8 (73%)
No children	1 (9%)
*Education*	
Skilled worker	6 (55%)
Unknown	5 (45%)
*Employment status*	
Employed	4 (36%)
Retired	3 (27%)
On leave because of partner’s illness	1 (9%)
On leave because of own illness	3 (27%)

Time from patient diagnosis to relative interview (weeks): Mean 27.4; range 7–50

### Recruitment and data collection

We invited relatives of the 26 patients included in the larger study. Two group interviews (n = 2 and n = 4 respectively) were conducted in a separate room at the OPS. In addition five spouses were interviewed individually as they were not able to attend the scheduled group interviews. The time from patient diagnosis to spouse interview varied from seven to fifty weeks. The themes explored were predetermined (influence on everyday life, procedures, the home, hygiene and the OPS) and formulated as five questions:

1. Try to give examples of how everyday life is experienced as a relative to a patient in the OPS?
2. Which things/tasks came with being a relative to a patient in the OPS?
3. What has it meant to your home that your spouse has been patient a in the OPS?
4. If I say the word ‘hygiene’, what do you think?
5. How do you, as a relative, experience the OPS concept?

All interviews were conducted by the first author, digitally recorded, and transcribed verbatim by a secretary.

### Setting

The course of treatment for the patients contains periods as inpatient and periods as outpatient where the patients live at home and appear at follow-up visits in the OPS every second day.

An important prerequisite for being managed in the OPS is that a relative is present at home all nights. Due to safety precautions patients have to measure temperature, take prophylactic antibiotics and record their wellbeing in diaries. All patients have a tunneled central venous catheter from which they or their close relatives draw blood samples, and do the cleaning routines of. Patients are instructed to avoid any contact with persons carrying an infection and avoid places where crowds gather. At home, the whole family should intensify the hygienic precautions and eschew mold.

### Analysis

The purpose of the inductive data analysis was to extract meaningful content from the experiences of the spouses, addressing the specific study aim. Data content analysis was an ongoing process performed in four steps as described by Miles and Huberman [12]. After reading the transcribed interviews text fragments were identified and descriptive codes were made which revealed patterns in the material that were condensed into descriptive categories. All interview data were organized, coded by the first author, but patterns and themes were continuously discussed with the other authors. Data were managed and retrieved using the computer program NVivo 10.

Sociological theories of everyday life constituted the analytical framework of this work [13]. This means that they were employed in the analytical process to open up and understand the descriptive categories emerging. Everyday life is in its simplicity ‘the life we live every day’, according to Bech-Jørgensen and it emerges from an interaction between everyday structure and circumstances and the ways people manage within these [13]. The everyday life and the surrounding world are largely taken for granted, as we create routines to be reproduced and repeated in continuous interaction with others, which also happens in the life of AL patients and their spouses. The life world is a shared ‘world of our common experiences’ extending beyond the individual [14]. We assume with this theoretical outset that the experience of patients managed in the OPS and their spouses are intertwined and construct a common life world. However, that life world takes on new structures and is continuously negotiated in the pervasive presence of AL and treatment, inseparable from the specific contexts of everyday environments in hospital and at home.

## Results

The profound challenge of OPM to the structure and routine of everyday life of the spouses was a predominant issue, deeply affecting the taken-for-granted in social life and family interactions. Accounts from the spouses addressing everyday life, were analyzed inductively forming four descriptive categories: responsibility, challenging partnership, the family, and social network. “Burden” was not an isolated theme as the feeling of burden appeared across the categories as a continuous aspect of OPM. Emotional issues seemed secondary to the illness, treatment and physical issues in the first part of the treatment course, in line with findings in a patient study [15].

The spouses noted that the changes, brought about by AL, were out of their control. This fundamental loss of control framed the existential experience of caregiving and living with a seriously ill person.

### Responsibility

Spouses were keen to act and assume responsibility and they experienced additional responsibility when the patient shifted status from inpatient to outpatient. It was described as a huge job to keep up with the precautions specified by the OPS. A key issue was the need for meticulous cleaning and removal of all potted plants.

‘I feel that I have a sense of responsibility–dammit I have to keep myself at bay not to develop OCD [Obsessive Compulsive Disorder] to bacteria… well, because I think, well if he [the patient] gets sick is it because my cleaning wasn’t meticulous enough.’

10. Individual interview. Spouse to a man with relapse of acute myeloid leukemia.

It was a demanding responsibility for the spouses to observe and react adequately to sudden symptoms of the patients, like fever or bleedings. Spouses and patients did not always agree on when to discuss symptoms with the hospital staff. At all hours they could call the Department to discuss a situation and ask for advice, but still they had to make independent decisions at home about the severity of the situation.

‘So they [the nurses] gave the advice that we should measure the temperature again before we went to bed, but by then it was late, almost 11.00 pm and I had imagined that I would go to bed. So, I ended up saying that in light of the circumstances he was fairly well, and then, well, we went to bed. I considered waking up at 3.00 am to repeat the measure [of the temperature]–but well, it went all right…’

6. Group interview. Spouse to a man with acute lymphoblastic leukemia.

The weight of the responsibility and the impact on everyday life was more extensive and burdensome than the spouses expected.

‘I think that’s just how it is. But it just really surprised me, how much responsibility you get. Tina [the nurse] had really told us a million times. It wasn’t because she hadn’t done what she could to warn me. […] I think they do what is possible to ensure you are equipped for the challenge–well equipped—really… You just have to chirp any kind of small concern, and they were ready for talking and helping. I think there’s also good support to be found in making it work. And she [the nurse] asked me several times–can you cope with it because otherwise he [the patient] is staying here [in hospital]. And that’s really where I think you answer yes without any idea of what you are really up to.’

10. Individual interview. Spouse to a man with relapse of acute myeloid leukemia.

It was remarkable that the weight of this responsibility surprised the spouses even though they felt well informed. This speaks to the pervasive impact of OPM on the psychosocial well-being of the spouses, where the structural demands of caregiving add additional burden to their everyday life in a longer lasting period (6 to 12 months).

### Challenging partnership

All spouses described how patients were more short-tempered and experienced sudden uncontrollable episodes of crying and how they tried to cope with these emotional changes.

‘I think, going through the procedures they [the patients] do, then you [the patients] can get a little–well–it takes less to–for you to get annoyed and such. You get more edgy and I think you can sense that. And that’s probably just how it is and then you just have to–well, hold your tongue’

8. Group interview. Spouse to a man with acute myeloid leukemia.

The extra precautions and the medical tasks (e.g. caring for the central venous catheter) often placed the spouses in a nurse-like role. This challenged their relationship when the patient did not behave as advised by the health care professionals.

‘They [the health care professionals] can’t be blamed that they have a self-willed patient who once in a while does things he is not allowed to because now his patience can’t be stretched any further. That is where we had the largest conflicts. It is when he has done something that he is not allowed to do, then I get all hysterical because… I’ll be damned if he gets ill again, gets some bacterial infection.’

10. Individual interview. Spouse to a man with relapse of acute myeloid leukemia.

The prescribed medication was considered an important and complex task administrated by the spouse. The other procedures were often more equally shared between patient and spouse, but the spouse expressed a strong desire that the patient would take on more of the effort to get things done.

‘That’s what Thomas [patient name] always says, “she will take care of it’–and I gladly do that, but I just don’t always want to–it’s just once in a while–then you become so full of it because then there are injections and then there is changing of this and that and then there are pills and it is a 100 times during that day–well, yeah, it’s not worse than that, but there is just so much, and then there is also just a bit with the blood sugar when steroids are given. It is just constantly every two or three hours something has to happen. And then just now and then you sigh deeply and say: Could you at least just once in a while try to take over a bit? ‘But are you tired of me?’ yes–no, that’s not what it’s about, right… But I’m also knocked over a bit… but then I take another round and try to steel myself again, right.’

6. Group interview. Spouse to a man with acute lymphoblastic leukemia.

Spouses experienced that patients were mostly unable to think about anything other than themselves and getting through their illness. This left the responsibility, the caretaking and the communication with their children in the hands of the spouses.

‘Because the first time she was picked up [by the ambulance] I remember, when she was lying on the stretcher and was about to enter the ambulance. Then you stand there beside her, and then she says -looking me deeply in the eyes, then she says: ‘Now you are the one to take care of our children’. It really trickled down my spine. And I think I have a fairly good and strong mind.’

7. Group interview. Spouse to a woman with relapse of acute myeloid leukemia.

The natural order of the partnership was challenged by the psychosocial changes of the patients. Furthermore, the caregiver role of the spouses created an unequal distribution of the responsibility and the medical tasks.

### The family

In families with young children the spouse felt torn between the patient and the children, whereas grown-up children were experienced as partners in managing the fear, thoughts and practical things like keeping the house and garden.

‘She [daughter] has also spent much time at home with me, whenever it was possible. And she wants to–also on weekends with her boyfriend, and they have all been like that. […] It was good to be together when dad was over there [at the hospital], right. […] That has been really good, because we’ve talked much about it with each other, all the time and every time something happened.’

11. Individual interview. Spouse to a man with acute myeloid leukemia.

With the patient at home, young children had to organize their play-dates according to the blood test results of the parent, as not to attract unnecessary infections to the house. Children were very alert to the movements and well-being of the ill parent, noticing and measuring the severity by little things, such as if they were present to read a bed time story.

‘Just when he [the patient] got home he was standing there looking at the stairs and I could hear how he sighed and fought to ascend the stairs. To him it was climbing of the highest caliber. [] Fourteen days later or something like that, then suddenly the oldest [boy] said: ‘Dad, have you recovered?’ [] And Johan [patient name] said no and asked why. ‘Well, you could follow me up the stairs this time’. So, he registered this–and he notices a lot.’

10. Individual interview. Spouse to a man with relapse of acute myeloid leukemia.

Relations in the family were however also strengthened during the trajectory of AL. The spouses were delighted and emphasized this as one of the few good things that followed from the disease.

‘Well, a trajectory like this also does some marvelous things, as Jacob [patient name] and his two siblings have developed the closest, most loving relationship that you can imagine. And now that he has also opened up to his grandchildren, really, he has never been caring much for the kids. The boys, the kids, our children, it’s always been mommy. […] Really, it’s a new world that’s opened up to him, I must say. A good world. A world where he really feels that he means a lot to the family if just he allows himself to.’

2. Individual interview. Spouse to a man with chronic myelomonocytic leukemia.

(Chronic myelomonocytic leukemia is a special kind of leukemia, which often turns into acute leukemia after short time. It is treated and managed like an acute leukemia, which is why these patients and relatives are a part of this study)

Despite all the hurdles appearing through the course of treatment spouses experienced that the relations of the family network were developed positively. The age of the children, however, seemed to be crucial to how the relation to the children was perceived.

### Social network

The patient was at the center of attention from everyone and his or her needs had first priority. All spouses found that self-evident, but as the course of treatment prolonged they could not keep neglecting their own needs. They expressed that family and friends were very important relations as a means for creating a personal space away from the patient and as an outlet to express their feelings and fears, which they did not want to share with the patient.

‘The ill gets constant care, the ill all the time gets: ‘Oh you are so good at this, and well, and are told how marvelous it is and so on and so forth’ but the one standing at the sideline… […] What I want to say is that it’s not particularly easy to be a relative. And then it is really good that you have a network, and you have … In my case not friends but my family.’

2. Individual interview. Spouse to a man with chronic myelomonocytic leukemia.

Having the patient at home with the precautions, that followed, affected the normal social life of the entire family. All members learned to live and act in accordance with the patients’ wellbeing and are very alert when blood counts were low. This often prevented having guests.

‘Actually, we don’t think that we have had many visits because, well first it was winter. And now with grandchildren, right, they catch all kinds of infections in school and kindergarten. […] And our daughter, she understood that very well and she didn’t think that they should come here if there was the least to worry about. And Christmas we celebrated by ourselves at home because, well his numbers were very low and then we wouldn’t risk anything. We had a nice Christmas on our own.’

5. Group interview. Spouse to a man with acute myeloid leukemia.

Furthermore, the spouses felt uncomfortable leaving the patient home alone. Feeling that it was unfair to the patient if they participated in social arrangements, they rarely did.

The spouses expressed a need of their social network for supportive reasons, but at the same time they were so conscientious to respect the precautions stated by the OPS that it hampered their social life.

## Discussion

During OPM the burden of the caregiver responsibility influenced the everyday life of spouses and challenged the partnership. Due to the restrictions of OPM, everyday life structures of the couples and families introduce new, strictly regulated routines that challenge the mater of course of everyday life. The order, repetitions, habits, routines and things that are taken for granted, which constituted family interactions before OPM is what is noted by theories of everyday life [13] as the fundamental condition for sustaining everyday life, as such maters of course prevent the need for constant evaluation and interpretation of phenomena in the world [13]. The disruption of everyday life order introduced by the OPM produces a need for constant evaluation and interpretation (of social interactions as well as symptoms related to treatment) that is highly demanding.

Spouses found it specifically burdensome to oversee and be constantly on call to assist the fragile AL patients in managing their treatment and expressed conflicting emotions of anxiety and exhaustion. The social life of the whole family was affected but illness also seemed to impact family relations developed positively.

The discussion addresses the issue of responsibility and the vigilance dominating everyday life of spouses to AL patients in the context of the individualization of the health care system in late modern society [16–18], where a larger part of responsibility for decisions and treatment is placed with the patients, and in the context of this study, their relatives. Next, we discuss how a lack of preparedness in spouses for the tasks and challenges in OPM may be adding to their sense of burden, pointing toward future areas of concerns for the clinical practice in OPM and education of patient and spouses to enhance empowerment. Finally, some study limitations are discussed.

As reflected in the results concerning responsibility and partnership this study illustrates how difficult it is for severely ill AL patients to meet the demand for accountability in treatment, as is a dominant trait of the health care system in the late modern society [16–18]. They were not able to take on the responsibility for the expected awareness of symptoms and all needed procedures leaving the spouses as the ones who actually manage the complex treatment at home. None of the spouses ever questioned or refused the caregiver role, although it was overwhelming and stressful. It seems that the spouses themselves expected to be responsible, active caregivers trying to live up to an unspoken late-modern ideal [19]. This speaks to a possible important point of difference between the information provided by health care professionals and the knowledge actually acquired and required by patient and spouse. However, patient and spouse may need to live with and experience OPM before they can truly understand how profoundly everyday life of the whole family is changed and challenged by the treatment.

As reflected in the results concerning social network, an invisible hyper-awareness was a consistent state of being alert 24/7, even when spouse and patient were apart. Such burdensome alertness and constantly monitoring are in line with findings in a previous interview study with 17 partners and daughters of chronic heart failure patients in an outpatient clinic [20]. It is usual to be alert in acute situations but with a patient suffering a long-term illness it becomes a more persistent state. Our findings suggest that such persistent alertness accounts for a large part of the experienced burden. They can never be relaxed when feeling responsible for their spouses’ life and the everyday life of their family.

The findings across this study, suggest that spouses did not feel well prepared to take on the tasks expected of them by patients, hospital and society. Empowerment is the sense of the positive outcome of the individualization in the health care system, but being put in control of treatment management is not equivalent to feeling in control of such management [21]. The concept of OPM does not include the possibility of a break or of refusal. Lack of choice and patients’ high motivation for this type of management may also add to the spouse’s sense of being overwhelmed and burdened [10].

Partners to AL patients represent individuals with different resources, responsibilities and life expectancies and will, to various degrees, be vulnerable to the challenges. A number of the AL patients are older and have a number of diseases and in general they may, along with families with children living at home, be the most vulnerable. The study underlines the need for professional support to the caregivers. Feelings of responsibility and burden may be reduced by a supportive talk with a health care professional [22]. At a time when OPM has become the state-of-the-art treatment for AL and is being introduced to other patient groups as well, development of educational practices in the clinic is important.

The limitations of this study include the use of interviews [11], which may have favored female spouses who were more keen to verbalize their thoughts and concerns. As only a fourth of participants were men the experiences of male caregivers may not be adequately reflected by our material. However, no gendered variation was seen across our material, when looking at the distribution of patterns and descriptive categories in the analysis. Further, the study is limited by the fact that half of the invited relatives turned down our invitation to participate. The most reported cause to the missing participation was lack of energy due to the overwhelming caregiver burden. This fact may have caused an underestimated experience of caregiving in our results. In general, this may have limited the perspective brought out in our material. However, our inductive analysis engaging a theoretical framework, opens up the possibility for an analytical generalization as is common in qualitative research with few participants [23] which makes it possible to draw out themes that may conceptually apply more to relatives of AL patients more broadly. All relatives included were spouses although a number of patients living alone fulfilled the OPS precaution by help from neighbors and friends. Experiences specific to being a non-partner relative may therefore have been overlooked.

## Conclusions

In conclusion, this study finds that the caregiver role is burdensome and with implications to the partnership and family life. The need for support varies over time, with the course of treatment and between individuals and families. Cancer treatment is an important issue in modern health care. OPM will increase for several groups of severely ill patients. This study suggests the need for proactive information and support to caregivers of intensively treated patients in OPM to ensure patients’ and the caregivers’ quality of life and satisfaction with care.

## Supporting information

S1 Fig Enrollment of relatives. (PPTX)
