# Supplementary material for: Living with outpatient management as spouse to intensively treated acute leukemia patients
Source: PLoS One. 2019 May 15;14(5):e0216821. doi: 10.1371/journal.pone.0216821 (PMC6519813; doi:10.1371/journal.pone.0216821)
Supplement: S1 Fig — (PPTX) [file pone.0216821.s001.pptx]

## Slide 1
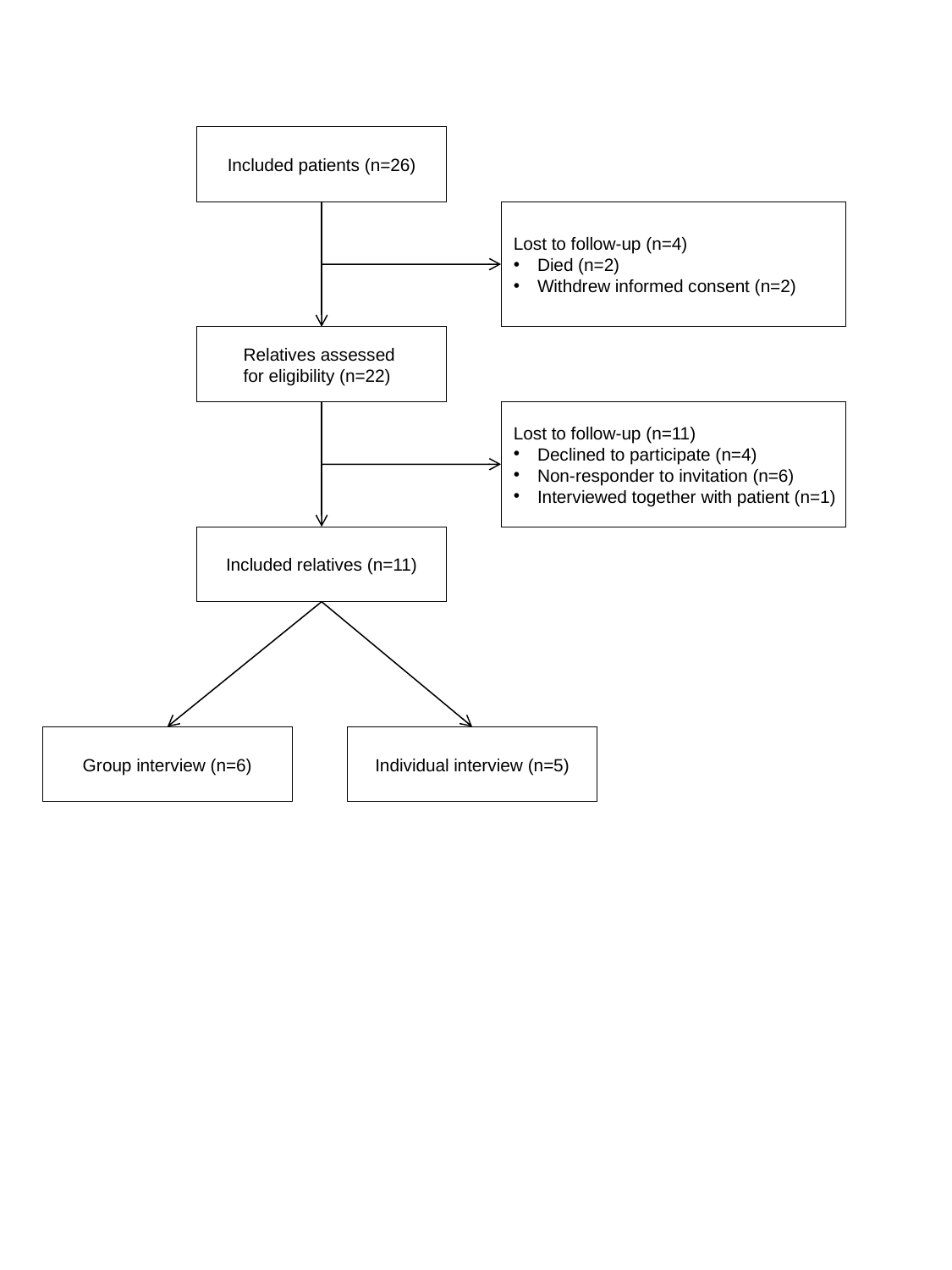

Included patients (n=26)
Lost to follow-up (n=4)
Died (n=2)
Withdrew informed consent (n=2)
Relatives assessed
for eligibility (n=22)
Lost to follow-up (n=11)
Declined to participate (n=4)
Non-responder to invitation (n=6)
Interviewed together with patient (n=1)
Included relatives (n=11)
Group interview (n=6)
Individual interview (n=5)
